# The complete chloroplast genome of *Hosta plantaginea* (Lam.) Aschers

**DOI:** 10.1080/23802359.2020.1823266

**Published:** 2020-09-22

**Authors:** Jian Dou, Hongjiang Wang, Mimi Li, Shengnan Huang, Haiying Tong

**Affiliations:** a Institute of Botany, Jiangsu Province and Chinese Academy of Sciences, Nanjing, China; b The Jiangsu Provincial Platform for Conservation and Utilization of Agricultural Germplasm, Nanjing, China

**Keywords:** *Hosta plantaginea* (Lam.) Aschers, Asparagaceae, chloroplast genome, Hosta

## Abstract

*Hosta plantaginea* is an important ornamental and horticultural plant endemic to China. In this study, we generated complete chloroplast genome of *H. plantaginea* using high-throughput sequencing. The complete chloroplast sequence is a circular molecule of 157,091 bp in size, consisting of a large single copy (LSC, 86,061 bp) and a small single copy (SSC; 18,282 bp) separated by a pair of inverted repeats (IRs; 26,374 bp), The total GC content is 37.8%, with 35.9, 31.7, and 43.0% in LSC, SSC, and IRs, respectively. A total of 132 genes are annotated, including 84 protein-coding genes, 38 tRNAs, 8 rRNAs, and 2 pseudogenes. The phylogenetic analysis revealed that *H. plantaginea* was formed to be the early diverging species within *Hosta*.

The genus *Hosta* belongs to the family Asparagaceae, and is a group of rhizomatous perennial herbs, which are widely cultivated as ornamental and horticultural plants. There are about 45 species distributed throughout the world and four of them are native to China, including *H. albofarinosa*, *H. ensata*, *H. plantaginea,* and *H. ventricosa*. All of them except *H. ventricosa* are endemic to China (Chen and Boufford 2000). *H. plantaginea* has high cultural, medicinal and ornamental value. The flowers are used for the treatment of sore throat in Mongolian medicine for hundreds of years (Li et al. 2012). The representative constituents, steroidal saponins and amaryllidaceae alkaloids, are proved to have anti-inflammatory, anti-tumor, and antimicrobial activities (He et al. 2016). *Hosta plantaginea* is considered to be the most valuable fragrant hosta species compared to the majority of *Hosta* (Liu et al. 2015). It combines many distinctive features such as fragrant large white flowers, late flowering, sprouting new foliage and withstanding hot-humid weather during the summer months. So it is an extremely important breeding germplasm resource. In this study, we present the complete chloroplast genome of *H. plantaginea* using Illumina sequencing technology. The results will be benefit for the new germplasm innovation of *Hosta*, as well as phylogenetic studies.

Leaf samples of *H. plantaginea* were collected from Nanjing Botanical Garden Memorial Sun Yat-Sen (32°3′19″N, 118°49′44″ E). Voucher specimen was stored at the Herbarium of Institute of Botany, Jiangsu Province and Chinese Academy of Sciences (NAS) under No. 06151698. The genomic DNA was isolated from the fresh leaves by a method of modified CTAB (Doyle and Doyle 1990) and subsequently sequenced on an Illumina Hiseq X-ten platform (San Diego, USA) at Novogene (Beijing, China). The raw paired-end (PE) reads were obtained for chloroplast genome assembly by NOVOPlasty 2.7.2 (Dierckxsens et al. 2017). The assembled genome was annotated via GeSeq (Tillich et al. 2017) and examined by manual in software Geneious 11.1.5 (Kearse et al. 2012).

The complete chloroplast (cp) sequence size of *H. plantaginea* was 1,57,091 bp in length, comprising a large single copy (LSC; 86,061 bp), a small single copy (SSC; 18,282 bp), separated by a pair of inverted repeats (IRs; 26,374 bp). The complete sequence was deposited in GenBank under the accession number MT810382. A total of 132 genes were identified, including 84 protein-coding genes, 38 transfer RNA genes (tRNA), 8 ribosomal RNA genes (rRNA) and 2 pseudogenes (ψ*inf*A and ψ*rps*16). Among them, 6 protein-coding genes, 8 tRNA genes, and 4 rRNA were repeated in IR regions. The percent GC content of *H. plantaginea* cp genome sequence was 37.8%.

The 11 complete cp genomes of Asparagaceae were aligned using MAFFT 7.409 (Katoh and Standley 2013). And phylogenomic reconstruction was generated based on whole cp sequences using Maximum likelihood analysis in RAxML (Stamatakis 2014) using *Yucca queretaroensis* (KX931468) and *Y. schidigera* (KX931469) as outgroups. As a result, *H. plantaginea* was formed to be the early diverging species with 100% bootstrap support (Figure 1).

**Figure 1.**
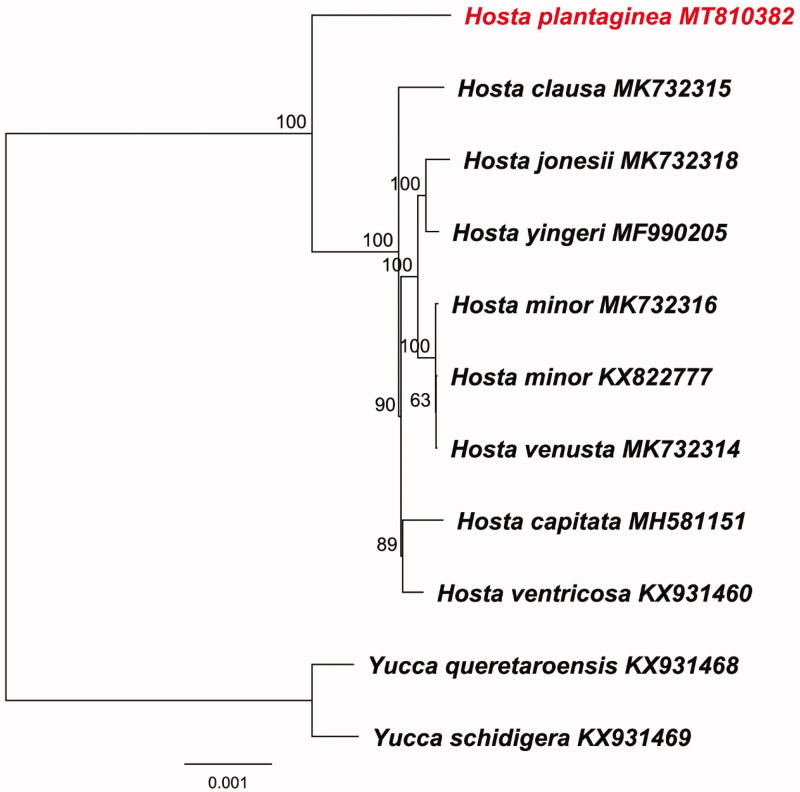
Phylogenomic reconstruction of *Hosta plantaginea* based on maximum likelihood (ML) of complete chloroplast genomes. The bootstrap values after 1000 replication are shown next to the nodes.

## Data Availability

The data that support the findings of this study are openly available in GenBank (https://www.ncbi.nlm.nih.gov) with the accession number is MT810382.
